# Missense substitutions in the BTB domain of ZBTB24 can lead to protein instability and cause ICF2 syndrome

**DOI:** 10.1093/hmg/ddaf182

**Published:** 2025-12-08

**Authors:** Or Givol, Ido S Han, Francesco Cecere, Liran Giladi, Noa Dotan-Glick, Revital Shemer, Atar Lev, Amos J Simon, Nivin Moustafa-Hawash, Chen Itzkovich, Vered Schichter-Konfino, Raz Somech, Karin Weiss, Daniel Kornitzer, Motoko Unoki, Maria Rosaria Matarazzo, Sara Selig

**Affiliations:** Pediatric Department, Hillel-Yaffe Medical Center, Hashalom Street, Hedera 38100, Israel; Department of Genetics and Developmental Biology, B. Rappaport Faculty of Medicine, Technion-I.I.T., 1 Efron Street, Haifa 31096, Israel; Department of Biomedical Sciences, Institute of Genetics and Biophysics "Adriano Buzzati-Traverso", Consiglio Nazionale delle Ricerche (CNR), Via Pietro Castellino 111, Naples 80131, Italy; Department of Genetics and Developmental Biology, B. Rappaport Faculty of Medicine, Technion-I.I.T., 1 Efron Street, Haifa 31096, Israel; Department of Genetics and Developmental Biology, B. Rappaport Faculty of Medicine, Technion-I.I.T., 1 Efron Street, Haifa 31096, Israel; Department of Genetics and Developmental Biology, B. Rappaport Faculty of Medicine, Technion-I.I.T., 1 Efron Street, Haifa 31096, Israel; Pediatric Department A and Immunology Service, Jeffrey Modell Foundation Center, Edmond and Lily Safra Children's Hospital, Sheba Medical Center, Tel Hashomer, Ramat-Gan 5262000, Israel; Pediatric Department A and Immunology Service, Jeffrey Modell Foundation Center, Edmond and Lily Safra Children's Hospital, Sheba Medical Center, Tel Hashomer, Ramat-Gan 5262000, Israel; Hemato-Immunology Unit, Hematology Lab and Sheba Cancer Research Center, Sheba Medical Center, Tel Hashomer, Ramat-Gan, 5262000, Israel; The Genetics Institute, Rambam Health Care Campus, 8 HaAliya HaShniya Street, Haifa 35254, Israel; The Clinical Research Institute, Rambam Health Care Campus, 8 HaAliya HaShniya Street, Haifa 352540, Israel; B. Rappaport Faculty of Medicine, Technion-I.I.T., 1 Efron Street, Haifa 31096, Israel; Pediatric Department, Hillel-Yaffe Medical Center, Hashalom Street, Hedera 38100, Israel; Allergy and Immunology Unit, Hillel-Yaffe Medical Center, Hashalom Street, Hedera 38100, Israel; Pediatric Department A and Immunology Service, Jeffrey Modell Foundation Center, Edmond and Lily Safra Children's Hospital, Sheba Medical Center, Tel Hashomer, Ramat-Gan 5262000, Israel; Gray Faculty of Medical and Health Sciences, Tel Aviv University, 35 Klatzkin Street, Tel Aviv 69978, Israel; The Genetics Institute, Rambam Health Care Campus, 8 HaAliya HaShniya Street, Haifa 35254, Israel; B. Rappaport Faculty of Medicine, Technion-I.I.T., 1 Efron Street, Haifa 31096, Israel; Department of Molecular Microbiology, B. Rappaport Faculty of Medicine, Technion-I.I.T., 1 Efron street, Haifa 31096, Israel; Department of Human Genetics, School of International Health, Graduate School of Medicine, The University of Tokyo, 7-3-1 Hongo, Bunkyo-ku, Tokyo 113-0033, Japan; Department of Biomedical Sciences, Institute of Genetics and Biophysics "Adriano Buzzati-Traverso", Consiglio Nazionale delle Ricerche (CNR), Via Pietro Castellino 111, Naples 80131, Italy; Department of Genetics and Developmental Biology, B. Rappaport Faculty of Medicine, Technion-I.I.T., 1 Efron Street, Haifa 31096, Israel; Laboratory of Molecular Medicine, Rambam Health Care Campus, 8 HaAliya HaShniya Street, Haifa352540, Israel

**Keywords:** ZBTB24, DNA methylation, BTB domain, ICF2 syndrome

## Abstract

ZBTB24 is a member of a protein family containing a Broad-Complex, Tramtrack, and Bric a Brac (BTB) domain, which functions in protein–protein interactions. ZBTB24, a transcription factor, binds its DNA targets through its C-terminal zinc finger (ZF) domain. Biallelic *ZBTB24* pathogenic variants lead to the rare autosomal recessive Immunodeficiency, Centromeric instability and Facial anomalies type 2 (ICF2) syndrome. The majority of ICF2 patients carry biallelic loss-of-function variants in *ZBTB24*. The remaining patients harbor missense variants in the ZF domain that compromise the ability of ZBTB24 to transcriptionally activate *CDCA7*, the gene responsible for ICF subtype 3 syndrome. Although an ICF2 patient with compound heterozygous pathogenic variants, including a missense variant (p.Ser59Gly) in the BTB domain, has been reported, no ICF2 patients with biallelic missense variants in any ZBTB24 domains other than the zinc finger domain have been described. Similar to all subtypes of ICF syndrome, *ZBTB24* pathogenic variants lead to significant DNA hypomethylation throughout the genome. Here we describe a patient with severe infections initiating during her first year of life, significant developmental delay and an abnormal facial shape, who carries a homozygous p.Val43Leu substitution in the BTB domain of ZBTB24. The patient’s peripheral blood cells demonstrate whole genome DNA hypomethylation with patterns identical to those found in verified ICF2 patients. Both the p.Val43Leu and p.Ser59Gly variants cause significant ZBTB24 protein instability. Thus, we demonstrate that pathogenic missense variants in the BTB domain of ZBTB24 can functionally act as loss-of-function variants that result in ICF2 syndrome.

## Introduction

DNA methylation is a major epigenetic modification of mammalian genomes that affects numerous cellular functions, including transcriptional regulation, silencing of transposable elements and chromosome X-inactivation in females. Abnormalities in establishment, reading and erasure of DNA methylation can lead to disease [Reviewed in [1–3]]. Defects in establishment and/or maintenance of DNA methylation characterize the rare but well-studied autosomal recessive disorder known as Immunodeficiency, Centromeric instability, and Facial anomalies (ICF) syndrome [2, 4]. ICF syndrome is genetically heterogeneous and biallelic pathogenic variants in *DNMT3B*, *ZBTB24*, *CDCA7*, and *HELLS* lead to ICF syndrome types 1–4 (OMIM: #242860, #614069, #616910, #616911), respectively [5–7]. One patient with atypical ICF syndrome was described with biallelic pathogenic variants in *UHRF1* [8]. DNMT3B is the major de novo DNA methyltransferase that methylates DNA during embryonic development, and participates throughout life in methylation maintenance [4]. ZBTB24 serves as a transcription factor that either silences or activates specific target genes [9, 10]. ZBTB24 positively regulates the expression of *CDCA7* [11], which forms a chromatin remodeling complex with HELLS. This complex recruits the DNMT1/UHRF1 maintenance DNA methylation machinery to newly synthesized DNA, particularly in genomic regions characterized by heterochromatin and late replication [12–15]. This ensures the maintenance of DNA methylation following DNA replication.

All ICF patients display abnormal DNA methylation landscapes in both unique and repetitive regions, with satellite 2 and 3 (sat 2 and sat 3) hypomethylation serving as a hallmark of all ICF syndrome subtypes [8, 12, 16]. Hypomethylation of these repeats is associated with decondensation of the pericentromeric regions in chromosomes 1, 9 and 16 and multiradial chromosomal structures involving these three chromosomes [17, 18]. Another common phenotype shared by the majority, but not all ICF patients is immunodeficiency, in which the main, but not exclusive, finding is agammaglobulinemia with existing B cells [19]. Facial anomalies, a prevalent phenotype among ICF patients, mainly include hypertelorism and a flat nose bridge [20, 21]. The severity of these phenotypes, as well as additional clinical and molecular phenotypes, varies among patients, partially depending on the affected gene. For example, the majority of ICF2 patients, carrying pathogenic variants in *ZBTB24*, display intellectual disability and motor developmental delay [20, 22].

ZBTB24, also known as PATZ2, is a member of a large family of ZBTB proteins that share in common a Broad-Complex, Tramtrack, and Bric a Brac (BTB) domain at their amino-terminus [23–25]. This approximately 120 residue-domain functions in protein–protein interactions, including homodimerization, heterodimerization and multimerization, with a strong preference for homodimerization [25]. BTB proteins perform highly diverse functions that are determined by their protein interactions via the BTB domain [23, 25]. Approximately 50 BTB proteins constitute the ZBTB protein family. These proteins contain a zinc finger (ZF) domain at their carboxy-terminus and serve as transcription regulators [24]. The ZBTB members bind their target DNA consensus sequences through the ZF domain, and regulate transcriptional activity through the interaction of their BTB domain with other proteins, enabling either activation or repression of their target genes [23]. In addition to its BTB domain and ZF domain composed of eight C_2_H_2_ motifs, ZBTB24 contains an AT-hook, a domain known to function in DNA binding [26]. ZBTB24 contributes to DNMT3B recruitment to its targets [10], and its recruitment to centromeric satellite DNA represses transcription at these sites [27]. An additional function attributed to ZBTB24 includes a regulatory role in non-homologous end joining, essential for immunoglobulin class-switch recombination and DNA double-strand break repair [28].

To date, approximately 40 ICF2 syndrome patients have been described in the literature [20, 29, 30]. The majority of these patients carry biallelic loss-of-function (LOF) variants in the form of nonsense or frame-shift variants. *ZBTB24* missense variants are less common in ICF2 patients, and only two patients have been reported with homozygous missense variants, p.Cys383Tyr and p.Cys408Gly, both of which disrupt one of the C_2_H_2_ motifs in the ZF domain [31, 32]. In addition, only one missense variant, p.Ser59Gly, located in the BTB domain, has been reported in a compound heterozygous ICF2 patient whose other *ZBTB24* allele carries a nonsense LOF variant [33]. As ZBTB24 transcriptionally activates *CDCA7* [9–11], all tested patients with *ZBTB24* null variants, or missense variants in the ZF domain show downregulation of *CDCA7* expression [34].

Here we describe a young girl with clinical manifestations including severe infections that initiated during her first year of life, developmental delay and abnormal facies. Exome analysis of this patient identified homozygosity for the sickle cell disease (SCD) causal variant in the *HBB* gene. Additionally, we detected a novel homozygous missense variant, p.Val43Leu, in the BTB domain of *ZBTB24*. We validated the pathogenicity of the *ZBTB24* variant by DNA methylation analysis, which demonstrated an ICF2-typical methylation pattern throughout the genome. Further molecular characterization indicates that this missense variant and the previously identified p.Ser59Gly variant render the ZBTB24 protein unstable. Thus, in addition to the ZF domain, missense variants in the BTB domain of ZBTB24 can function *de facto* as LOF variants and lead to ICF2 syndrome.

## Results

### Clinical presentation

**Figure 1:**
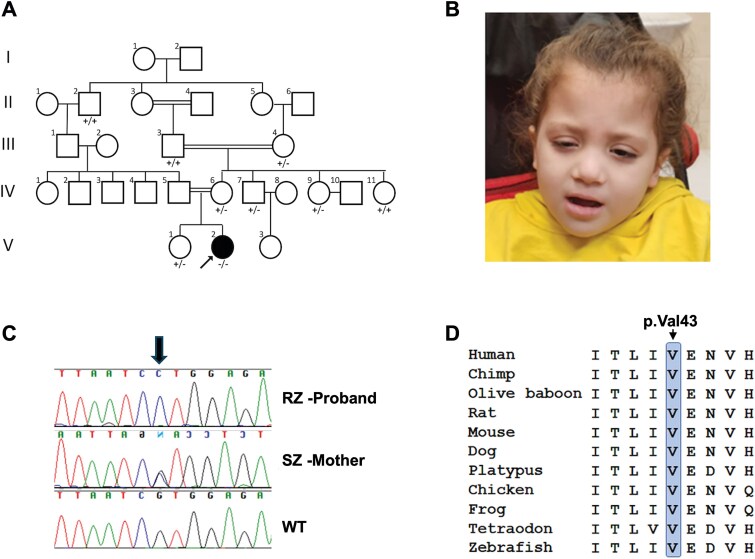
A novel missense variant detected in a conserved amino acid in the BTB domain of ZBTB24. A. Family pedigree of proband RZ who carries the novel variant in ZBTB24. The carrier status of ZBTB24:c.127G > C is depicted under the symbols of family members tested for this variation. +/+ does not carry the variant, +/− is a heterozygote carrier, −/− is a homozygous carrier. Arrow points to proband homozygous for this variant. B. Facial photograph of patient RZ. Note the hypertelorism and a flat nose bridge. C. Validation of *ZBTB24*: c.127G > C;p.Val43Leu variant by Sanger sequencing. Whole blood DNA from proband RZ, mother SZ, and a WT control were sequenced. Arrow points to the position of the variant. D. Evolutionary conservation of valine at position 43 in ZBTB24 (depicted by the blue strip).

The proband, a female designated patient RZ, was born at gestational age 38 + 2 weeks weighting 2770 grams, with APGAR scores of 9 and 10 at 1 and 5 min, respectively. Her parents are second degree cousins of Arab Muslim descent (Fig. 1A). After birth she was diagnosed with a recto-vestibular fistula. RZ developed neonatal jaundice on her first day of life that was treated with phototherapy, and workup showed no evidence of haemolysis.

At the age of 6 months, RZ was hospitalized due to suspected seizures. Workup was normal except for normocytic anemia. At the age of 10 months, she was admitted again due to fever, and was diagnosed with bacterial meningitis due to *Streptococcus pneumoniae*. On her fourth day of hospitalization RZ developed refractory status epilepticus and was transferred to the pediatric intensive care unit. Further neuroimaging were negative for malformations. Preliminary workup was noted for no neutropenia and no clear abnormality in levels of her gamma-globulins (Table 1). Gradually she improved, and was discharged with prophylactic treatment with Amoxicillin and Levetiracetam. To note, RZ received all her immunizations according to the Israeli protocol, which included two doses of 13-Valent pneumococcal conjugate vaccine (PCV13) up to the age of 4 months and a third dose at the age of 12 months.

**Table 1:** Hemoglobin fractions, complement and immunoglobulin levels in patient RZ.

	**6 months**	**10 months**	**1 year and** **3 months**	**1 year and** **6 months**	**1 year** **and** **10 months**	**1 year** **and** **11 months**	**3 years and** **9 months**	**3 years and** **10 months**	**5 years**	**5 years and** **4 months**
**Hb electrophoresis:**										
**Hemoglobin S**				67%			35%			
**Hemoglobin A**				9% (96–98%)			^a^46%			
**Hemoglobin F**				21% (<2%)			^a^16%			
**Blood smear:**										
**Schistocytes**	Neg		Neg	Pos	Neg			Pos	Neg	
**Sickle cells**	Neg		Neg	Neg	Pos			Pos	Neg	
**Howell Jolly bodies**	Neg		Neg	Pos	Neg			Neg	Pos	
**Spherocytes**	Neg		Neg	Neg	Neg			Neg	Neg	
**Immunoglobulins‡:**										
**IgE Total (IU/ml)**		< 4.86 (0.76–7.31)					< 4.86 (0.19–16.9)			
**IgM** **(mg/dL)**		39.5 (41–149)	40 (43–173)	31 (43–173)		57.1 (43–173)	60 (47–200)		29 (43–154)	40 (43–154)
**IgG** **(mg/dL)**		629 (294–1069)	1004 (345–1213)	864 (345–1213)		824.0 (345–1213)	958 (441–1135)		918 (436–1236)	1193 (436–1236)
**IgG Subclass 1 (mg/dL)**				760 (230–710)						
**IgG Subclass 2 (mg/dL)**				75.7 (30–170)						
**IgG Subclass 3 (mg/dL)**				108 (11–98)						
**IgG Subclass 4 (mg/dL)**		2.13 (6–63)		1.2 (4–43)						
**IgA** **(mg/dL)**		91.7 (16–84)	69 (14–106)	124 (14–106)		111.0 (14–106)	373 (22–159)		152 (25–154)	188 (25–154)
**HBV serology:**										
**HBcAb**					Neg					
**HBsAb (IU/L)**					9					
**HBsAg**					Neg					
**Vaccinations**										
**Diphtheria Ab (IU/ml)**								1.02		
**Measles Virus IgG (IU/ml)**						1.43 (Positive)	0.63 (Borderline)			
**Complement:**										
**C1q (mg/dL)**			25 (< 20.8)							
**C2 (mg/dL)**			>3.6							
**C3 (mg/dL)**		151 (90–180)	150 (90–180)							
**C4 (mg/dL)**		31.3 (10–40)	40 (10–40)							
**C5 (mg/dL)**			16.1							
**C6 (mg/dL)**			>12							
**C7 (mg/dL)**			8.7							
**C8 (mg/dL)**			16.9							
**C9 (mg/dL)**			42.3							
**Miscellaneous tests:**										
**Haptoglobin** **(mq g/dL)**		252 (20–300)	<10 (20–300)	<10 (20–300)	<10 (20–300)					< 10 (20–300)
**DAT** ^b^		Neg	Neg	Neg	Neg					
**Reticulocytes %**			24% [1–5]	10.3% [1–5]	16.8% [1–5]				9.1% [1–5]	
**G6PD levels** **(IU/g Hb)**				27.9						

^a^Hemoglobin electrophoresis was conducted shortly after red blood cells infusion.

^b^DAT – Direct Anti-globulin Test

At the age of 1 year and 3 months, RZ was admitted once again to the pediatric department, with a working diagnosis of sepsis and intravascular hemolytic anemia (due to high LDH and reticulocytosis and undetected haptoglobin levels). Her immunoglobulin levels demonstrated normal levels of IgA and IgG isotypes and her IgM level was slightly below the lower limit of the normal range (Table 1). Her blood culture was positive for *S. pneumoniae*. She was treated with Ceftriaxone and improved. After this hospitalization, and with ongoing prophylactic Amoxicillin treatment she did not experience additional severe bacterial infections.

RZ continued ambulatory workup for her hemolytic anemia and suspected immune deficiency. At age of 1 year and 6 months, hemoglobin electrophoresis demonstrated 67% hemoglobin S, 21% hemoglobin F and 9% hemoglobin A (Table 1), confirming a diagnosis of sickle cell anemia. She underwent spleen mapping with Tc-99 which demonstrated no uptake at all segments, and spleen ultrasound that demonstrated normal size and texture. Together, this imaging was diagnostic for complete functional asplenia. Further family history revealed sickle cell anemia in RZ’s maternal grandmother (III-4, Fig. 1A). RZ’s immunological workup included normal complement levels (C1–9) (Table 1). Her immunoglobulin levels had a repetitive pattern of levels within the normal limits for isotypes IgA and IgG, and IgM levels that were slightly below the lower limit of the normal range (Table 1). Serum protein electrophoresis demonstrated a normal pattern without any signs of monoclonal gammopathy (Table 1 and Supplementary Fig. 1). During the period between age 1 year and 10 month to 3 years and 10 months, a normal response to vaccinations was seen (Table 1).

On repeated physical examinations RZ demonstrated facial dysmorphism including hypertelorism, epicanthal folds and a flat nose bridge (Fig. 1B). RZ attends a special education preschool and is treated at a child developmental center due to global developmental delay.

### Exome analysis

Due to two severe pneumococcal infections during infancy, and the absence of indications for functional asplenia or auto splenectomy at that time, an inborn error of immunity (IEI) was suspected. To explore this possibility, exome sequencing was performed on blood DNA from patient RZ. This analysis identified a homozygous pathogenic missense variant in *HBB* (RefSeq NM_000518.5:c.20A > T;p.Glu7Val), confirming sickle cell disease (SCD, OMIM: #603903). An additional homozygous missense variant of uncertain significance was identified in *ZBTB24* (RefSeq NM_014797.3:c.127G > C;p.Val43Leu), which was validated by Sanger sequencing (Fig. 1C). Bi-allelic pathogenic variants in *ZBTB24* lead to autosomal recessive Immunodeficiency, Centromeric Instability and Facial Anomalies syndrome 2 (ICF2, OMIM: #614069). Segregation analysis of several family members, of which none displayed severe infections nor severe anemia, found no homozygotes for this variant (Fig. 1A), including RZ’s mother (SZ) and RZ’s sister, who are heterozygous for this *ZBTB24* variant. RZ’s father was not available for testing.

The Valine at position 43 in ZBTB24 is highly conserved across fish, amphibians, and mammals (Fig. 1D), and the c.127G > C variant is rare and not reported in the gnomAD database (version v4.1). SCD could explain the severe infections that RZ experienced during infancy, however, typically they would be expected to initiate later in childhood. Furthermore, her developmental delay and dysmorphism are beyond the phenotypic scope of SCD, and characteristic of ICF2 syndrome [22]. We, therefore, continued to explore the pathogenicity of the ZBTB24 p.Val43Leu variant.

### An ICF2-typical pattern of whole genome hypomethylation is found in patient RZ

**Figure 2:**
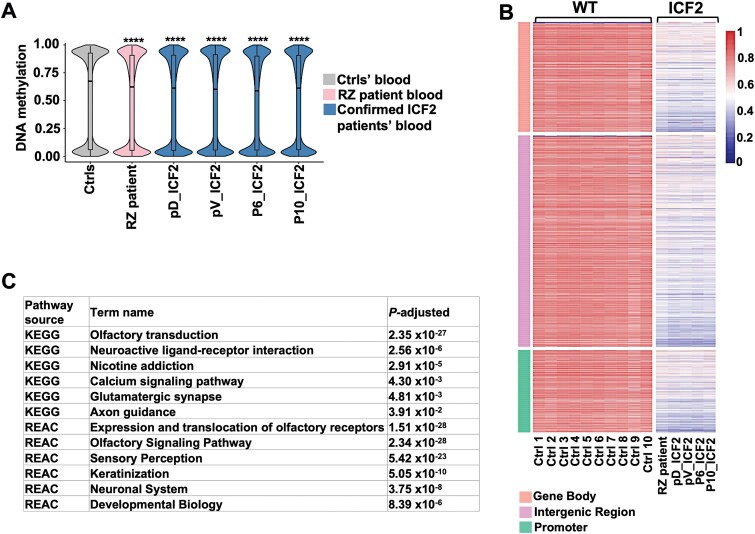
Whole genome DNA methylation profiling of RZ blood DNA confirms a typical ICF2 pattern of hypomethylation. A. A violin plot showing the distribution of whole-genome DNA methylation levels (expressed as β-values from 0 to 1) in control (Ctrls) (average of 10 control blood samples), RZ patient and four confirmed ICF2 patients (pV, pD, P6 and P10). *P*-adjusted values represent the Bonferroni-corrected *P*-values obtained from a two-sample Wilcoxon test with two-sided alternatives. ^****^indicates *P*-value < 0.0001. B. A heatmap depicting the DNA methylation levels of 2183 DMPs identified in RZ patient and the additional ICF2 patients in comparison to control blood samples. The genomic features of DMPs, i.e gene body, intergenic region or promoter associated, are highlighted as row annotations. C. Gene ontology analysis of 622 genes annotated to DMPs identified in RZ patient compared to the average of control blood samples. The 12 most significantly enriched KEGG and REACTOME pathways and the corresponding adjusted *P*-values (Benjamini-Hochberg FDR < 0.05) are shown in the table.

All ICF syndrome patients display hypomethylation throughout their genome [8, 12]. To determine the DNA methylation patterns of patient RZ, we performed whole genome DNA methylation analysis on patient whole blood DNA using the Infinium Methylation EPIC 850 K Bead Chip. We compared the findings obtained from RZ’s whole blood with those previously described from 10 wild type (WT) controls and four confirmed ICF2 patient blood samples [12]. The ICF2 patients include two siblings, pV and pD (who carry homozygous *ZBTB24* frameshift variants) [35], P6 (who carries a homozygous missense variant in the ZF domain of *ZBTB24*) [32], and P10 (who carries a homozygous *ZBTB24* nonsense variant) [6]. This analysis clearly demonstrated that patient RZ’s blood DNA displays significant global hypomethylation compared to the average of the 10 control samples, with a pattern highly similar to the four confirmed ICF2 patients (Fig. 2A). Overall, we identified 2183 differentially methylated probes (DMPs) in RZ’s whole blood, of which 1109 are positioned in intergenic regions, while 643 and 431 are associated with gene bodies and promoters of 622 genes, respectively (Fig. 2B). Thus, the DMPs throughout the genome of RZ are highly similar to those of other confirmed ICF2 patients.

To further compare RZ to the other ICF2 patients, we analyzed the gene ontology of the 622 genes annotated to DMPs (Fig. 2C and Supplementary Table 1). We found that the pathways significantly enriched among the hypomethylated genes in RZ are highly similar to those described in the confirmed ICF2 blood samples [12]. Notably, the majority of them are related to nervous system functions, which corresponds with the neurodevelopmental delay characteristic of ICF2 patients, and described also for RZ.

### Centromeric and pericentromeric satellite repeats are hypomethylated in RZ patient

**Figure 3:**
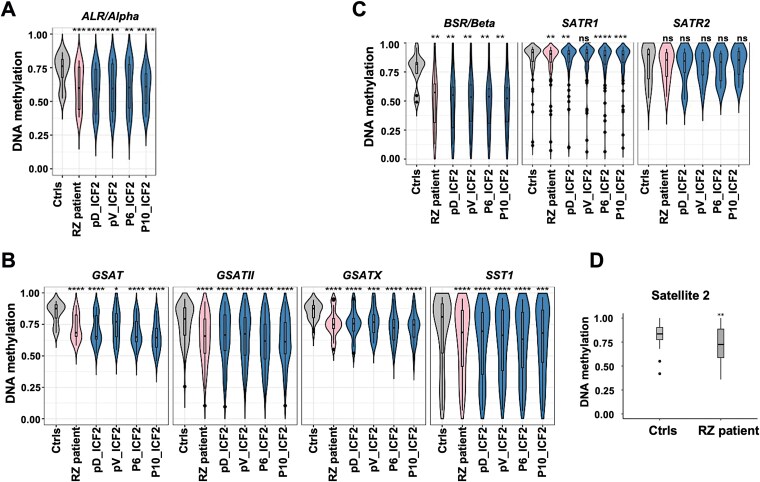
Patient RZ displays ICF2-typical hypomethylation at satellite repetitive regions. A—C violin plots showing the distribution of methylation levels of satellite repeats localized in (A) centromeric (ALR/alpha), (B) pericentromeric (GSAT, GSATII, GSATX, SST1) and (C) other genomic regions (BSR/Beta, SATR1, SATR2) in control (Ctrls) (average of 10 control samples), patient RZ and confirmed ICF2 pV, pD, P6 and P10 patients whole blood DNA. D. Boxplot displaying DNA methylation levels of satellite 2 (sat 2) repeats localized to chromosomes 1, 10 and 16 measured by region-specific bisulfite Sanger sequencing in control and RZ blood samples. *P*-adjusted values represent the Bonferroni-corrected *P*-values obtained from a two-sample Wilcoxon test with two-sided alternatives. ^**, ***, ****^ indicate *P*-values of < 0.01, < 0.001, < 0.0001, respectively. ns-Not significant.

A major hallmark of ICF syndrome is hypomethylation of pericentromeric satellite 2 and 3 (sat 2 and sat 3) repeats [12, 22]. In all ICF syndrome subtypes, besides ICF1, centromeric α satellite (α sat) repeats are additionally hypomethylated, as well as various other repeats [12]. To determine whether RZ patient displays this molecular phenotype, we analyzed CpGs on the methylation array positioned within satellite repeat regions which include centromeric repeats (ALR/Alpha), pericentromeric repeats (GSAT, GSATII, GSATX, SST1) and repeats dispersed across the genome (BSR/Beta, SATR1, SATR2) (Fig. 3A-C; Supplementary Table 1). This analysis revealed that all repeats hypomethylated in the ICF2 samples were also significantly hypomethylated in RZ, including α sat (ALR/Alpha) repeats.

Because sat 2 and sat 3 repeats are poorly represented on the Infinium methylation array, we performed region-specific bisulfite Sanger sequencing on RZ’s whole blood DNA and on two control blood samples to determine the DNA methylation status of sat 2 repeats. Based on a Human BLAT search (UCSC) of the PCR primers we utilized for this analysis, we amplified pericentromeric sat 2 repeats from chromosomes 1, 10 and 16, regions that are not covered by the methylation array (Supplementary Table 1). This analysis demonstrated that sat 2 pericentromeric repeats are also hypomethylated in RZ’s blood DNA (Fig. 3D). Thus, the methylation analyses strongly support that patient RZ has ICF2 syndrome in addition to SCD.

### Chromosomes prepared from RZ patient blood show an atypical phenotype

Pericentromeric repeat hypomethylation is associated with characteristic aberrations in white blood cell-derived metaphases in all ICF syndrome subtypes. These aberrations include decondensed pericentromeric regions and multiradial configurations involving chromosomes 1, 9 and 16 [17, 36, 37]. To determine whether RZ presents this phenotype, we prepared G-banded metaphase spreads from her blood. Examination of 117 metaphases failed to reveal the ICF syndrome typical chromosomal aberrations. However, notably, premature sister chromatid separation (PSCS) was evident in 6.8% of her examined metaphase spreads (eight out of the 117 metaphases) (Supplementary Fig. 2A). In contrast, in at least 132 metaphases from each of three WT control blood samples, and all together in 470 metaphase spreads, we found no evidence for PSCS. Interestingly, PSCS occurs in all chromosomes and not only in chromosomes 1, 9, and 16. Such a chromosomal phenotype, which implies centromeric malfunction, has not previously been described to our knowledge in patients with ICF syndrome of any subtype, or linked to epigenetic disturbances at centromeres.

To futher explore the cytogenetic phenotype of RZ’s cells, we expanded our cytogenetic analysis to a lymphoblastoid cell line (LCL) derived from RZ’s blood. We first analyzed the whole genome DNA methylation status of RZ LCLs. Similar to her blood sample, RZ LCLs were significantly hypomethylated compared to WT LCLs. However, the hypomethylation was less severe compared to that found in pD and pV LCLs, derived from confirmed ICF2 patients [35] (Supplementary Fig. 3). Examining the DMPs identified in RZ’s blood DNA, we found that the hypomethylation in her LCLs was less pronounced compared to her blood DNA. Nevertheless, the hypomethylation was significant compared to WT LCLs, and the methylation pattern was similar to those of the two other ICF2 LCLs (Supplementary Fig. 3).

After confirming the ICF2-typical hypomethylated status of RZ LCLs, we prepared G-banded metaphase spreads from RZ and pD LCLs. None of the metaphase spreads in either RZ or pD LCLs showed PSCS. However, in contrast to the blood derived metaphases, in 7.7% of the examined metaphase spreads of RZ (eight out of 103) the pericentromeric region of one copy of chromosome 1 was decondensed (Supplementary Fig. 2B). In pD LCLs, 11.8% (13 out of 110) showed a similar phenotype. Thus, the LCLs derived from RZ’s blood show a typical ICF-related cytogenetic phenotype.

### Pathogenic missense variants in the BTB domain decrease full length ZBTB24

**Figure 4:**
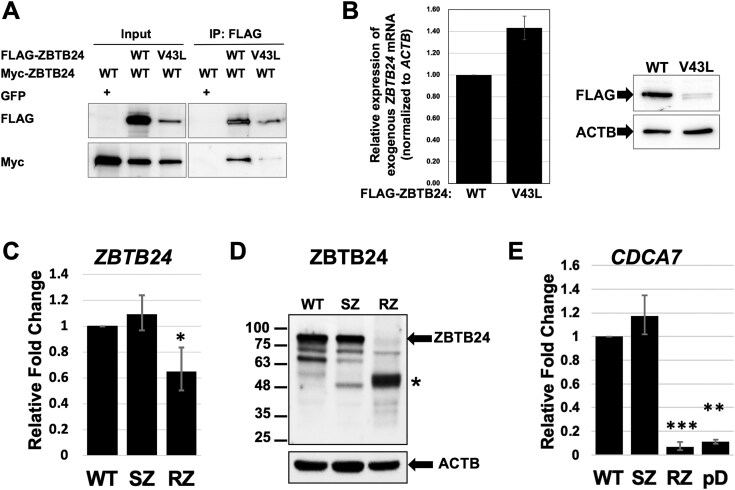
The p.V43L missense variant in the BTB domain decreases full-length ZBTB24. A. Myc-ZBTB24_WT_ was co-expressed with FLAG-ZBTB24_WT_ or FLAG-ZBTB24_V43L_ in HEK293T cells. As a control, Myc-ZBTB24_WT_ was co-expressed with GFP. Immunoprecipitation was performed using anti-FLAG affinity gel. While Myc-ZBTB24_WT_ was efficiently coimmunoprecipitated with FLAG-ZBTB24_WT_, expression levels of FLAG-ZBTB24_V43L_ were very low in the input, which made it impossible to assess binding affinity between Myc-ZBTB24_WT_ and FLAG-ZBTB24_V43L_. B. Expression levels of FLAG-ZBTB24_WT_ or FLAG-ZBTB24_V43L_ in HEK293T cells were examined at RNA level by RT-qPCR (left) and at protein level by western blot analysis (right) forty-eight hours after plasmid transfection. While mRNA levels are comparable between *FLAG-ZBTB24*_*W*T_ and *FLAG-ZBTB24_V43L_*, protein levels of FLAG-ZBTB24_V43L_ are very low compared to FLAG-ZBTB24_WT_. Accurate protein loading was verified using β-actin (ACTB) protein levels as a control. C. Expression levels of *ZBTB24* were assessed in WT (control GM18486), SZ and RZ LCLs by RT-qPCR, normalized to β-actin and presented as fold change relative to expression levels in WT LCLs. RT-qPCR was performed twice for each of two biological repeats. Error bars represent standard errors. *P*-values were determined by Student’s t-test (^*^p < 0.05). D. Western blot analysis of ZBTB24 expression in WT (control GM18486), SZ, and RZ LCLs. Upper arrow points to the position of the main ZBTB24 isoform. Asterisk marks a possible degradation product. Accurate protein loading was verified using β-actin (ACTB) protein levels as a control. Size markers in kDa appear on the left. E. Expression levels of *CDCA7* were assessed in WT (control GM18486), SZ, RZ and pD LCLs by RT-qPCR, normalized to β-actin and presented as fold change relative to expression levels in WT LCLs. RT-qPCR was performed twice for each of two biological repeats. Error bars represent standard errors. *P*-values were determined by Student’s t-test ( ^**^p < 0.01, ^***^p < 0.001).

The N-terminal BTB domain, shared by the ZBTB-family members, is crucial for homodimerization [38–40]. Specifically, the BTB domain of ZBTB24 was shown to homodimerize, as well as heterodimerize with ZBTB19 (PATZ1) [40]. Based on this evidence, we hypothesized that the Val-to-Leu amino-acid substitution in the BTB domain of RZ’s ZBTB24 might impair its dimerization ability. To extend these previous analyses, we first sought to determine whether the full-length ZBTB24 protein also homodimerizes. By IP-western blot analysis, we found that Myc-ZBTB24_WT_ was efficiently coimmunoprecipitated with FLAG-ZBTB24_WT_, suggesting that full-length ZBTB24_WT_ indeed forms homodimers (Fig. 4A). Interestingly, despite transfecting equal amounts of pFLAG-ZBTB24_WT_ or pFLAG-ZBTB24_V43L_ with pMyc-ZBTB24_WT_, the FLAG-ZBTB24_V43L_ protein levels were significantly lower than FLAG-ZBTB24_WT_ levels (Fig. 4A, input), suggesting that ZBTB24_V43L_ may be unstable. Consequently, only a small amount of Myc-ZBTB24_WT_ was coimmunoprecipitated with FLAG-ZBTB24_V43L_, making it challenging to assess whether the Val-to-Leu substitution compromises ZBTB24 dimerization.

To explore the cause for the low levels of FLAG-ZBTB24_V34L_ protein, we transfected HEK293T cells with pFLAG-ZBTB24_WT_ or with pFLAG-ZBTB24_V43L_ and examined the transcript and protein levels of the exogenous ZBTB24_WT_ and ZBTB24_V43L_. We found that despite slightly higher mRNA levels of FLAG-ZBTB24_V43L_ compared to FLAG-ZBTB24_WT_, the protein level of full-length FLAG-ZBTB24_V43L_ was considerably lower than that of FLAG-ZBTB24_WT_ protein (Fig. 4B). Thus, we concluded that the low levels of FLAG-ZBTB24_V43L_ are due to protein instability. Notably, the protein level of full-length FLAG-ZBTB24 containing the BTB missense variant p.S59G, described in a compound heterozygous ICF2 patient [33] was also significantly reduced compared to ZBTB24_WT_ (Supplementary Fig. 4). This indicates that at least one additional pathogenic missense variant in close vicinity to p.V43L, similarly renders the protein unstable.

### The endogenous full-length ZBTB24-V43L protein is decreased in patient RZ derived lymphoblastoid cells

We next explored the expression levels of the endogenous ZBTB24-V43L protein. The only available patient-derived cells to test this were the LCLs derived from whole blood of RZ. We first determined the mRNA level of *ZBTB24* in LCLs derived from a WT control, from SZ (RZ’s mother—a heterozygous carrier of the c.127G > C [p.V43L] variant), and RZ. Analyses of *ZBTB24* mRNA levels demonstrated that SZ LCLs express normal levels, while in RZ LCLs the levels were reduced at a borderline significant level (P value = 0.042, Fig. 4C). In contrast, at the protein level, full length ZBTB24 protein was almost completely absent in RZ LCLs (Fig. 4D). As expected, SZ LCLs showed intermediate levels of full length ZBTB24. A faster migrating protein product of approximately 50 kDa was evident in both SZ and RZ LCLs, with its level significantly higher in RZ compared to SZ (Fig. 4D). Since the ZBTB24 antibody recognizes the C-terminus of the protein, unlike the FLAG antibody that recognizes the N-terminal tag of the exogenously expressed ZBTB24 (Fig. 4A and B), this protein product could represent a C-terminal degradation product of ZBTB24. Based on its molecular weight, this C-terminal product lacks both the BTB domain and the AT-hook. To determine whether this shorter protein can carry out its function in transcription activation, we determined mRNA levels of *CDCA7*, one of ZBTB24 targets, and the gene mutated in ICF3 syndrome [7]. As demonstrated previously in ICF2 LCLs [12], and as repeated here for pD LCLs, the expression level of *CDCA7* mRNA in RZ LCLs was significantly lower compared to WT or SZ LCLs (Fig. 4E). Similar to *CDCA7*, the transcription of another ZBTB24 target, *RNF187* [27], was downregulated in RZ LCLs (Supplementary Fig. 5), further supporting the loss of the ability of the mutated ZBTB24 to activate its targets.

To assess whether the smaller-sized protein product is the consequence of partial protein degradation mediated in a proteasome-dependent manner, we treated patient RZ LCLs with the proteasome inhibitor MG-132 and found that the level of the endogenous mutated full-length ZBTB24 was only marginally increased (Supplementary Fig. 6A). The amount of the exogenous mutated full-length FLAG-ZBTB24 proteins was even less affected by MG132 and another proteasome inhibitor, Bortezomib, compared to the endogenous mutated ZBTB24 (Supplementary Fig. 6B). In summary, we could conclude that the patient-derived BTB missense variant results in a shortened ZBTB24 protein lacking the BTB domain and the AT-hook, which is presumably dysfunctional. Whether the proteasome machinery is involved in this process could not be inferred with certainty. To investigate whether the mutated protein is degraded via the autophagy pathway, we treated SZ and RZ LCLs with Bafilomycin A1 (BafA), an inhibitor of autophagy, and examined the stability of ZBTB24. Although the change in ratio between LC3I and LC3II indicated that indeed autophagy was blocked following the treatment with BafA, we could not observe any stabilization of ZBTB24 in RZ LCLs (Supplementary Fig. 7). In the carrier, mother SZ, moderate stabilization of ZBTB24 was apparent, presumably originating from the normal *ZBTB24* allele. Interestingly, following the treatment with BafA the approximately 50kD protein product was not visualized in neither the mother nor the patient LCLs.

## Discussion

In this study we report a female patient, named as RZ, who carries a homozygous novel missense substitution, p.V43L, in the BTB domain of ZBTB24, the causative gene for ICF2 syndrome [6]. Approximately 40 patients with ICF2 have been reported to date, the majority of them carrying nonsense or frameshift variants in *ZBTB24*. The remaining have missense variants in the ZF domain [20]. Essentially, the ZF domain is dysfunctional in all previously reported ICF2 patients. ZBTB24 LOF leads to compromised transcriptional activation of *CDCA7*, one of ZBTB24 targets [9, 10], and the causative gene for ICF3 syndrome [7]. Here, we demonstrated that missense variants in the BTB domain can also lead to ZBTB24 LOF by leading to the degradation of the full length protein, and thus abolishing the transcription of *CDCA7*, as well as other targets of ZBTB24. In addition to the homozygous variant in *ZBTB24*, exome analysis of patient RZ revealed homozygosity for the SCD variant in the *HBB* gene. However, SCD alone cannot explain several of the patient’s phenotypes, such as the facial dysmorphism and global developmental delay, as well as the uncommon invasive pneumococcal infections [41, 42]. The majority of ICF2 patients display intellectual disability to variable degrees, and frequently also motor developmental delay [19, 22]. However, these later phenotypes are common to many genetic syndromes, and cannot affirm by themselves the occurrence of ICF2 syndrome. The most compelling evidence that supports the occurrence of ICF2 in patient RZ, is the observed changes in DNA methylation. All five types of ICF patients manifest hypomethylation of pericentromeric repeats [8, 12], and whole genome hypomethylation patterns are highly similar between ICF1–4 subtypes. However, ICF1 is distinguishable from ICF2, 3 and 4 [12, 43], and one patient with atypical ICF syndrome, carrying mutations in *UHRF1*, has a distinctly patterned hypomethylated genome [8]. When we analyzed DNA methylation throughout the genome of patient RZ, the patterns in peripheral blood cells were identical to those of the other bona fide ICF2 patients (Figs 2 and 3), including at the classically affected repetitive regions. Additionally, LCLs derived from RZ’s blood demonstrated decondensation of the pericentromeric region of chromosome 1, an ICF-typical cytogenetic abnormality. The presence of an ICF2 DNA methylation signature in RZ, in concert with her immunodeficiency, the cytogenetic abnormalities, abnormal facial shape and global developmental delay, strongly support the pathogenicity of the ZBTB24-V43L variant. Our finding that the p.V43L variant renders ZBTB24 highly unstable, similar to the additional p.S59G variant detected in another compound heterozygote ICF2 patient, adds another degree of confidence regarding the causality of the BTB missense variants in eliciting this syndrome.

The molecular verification of ICF2 syndrome in this patient expands the cellular and clinical variability of ICF syndrome. With relation to the cytogenetic markers, this study indicates that pericentromeric decondensation and formation of multiradial chromosomes, defined as hallmarks of ICF syndrome, are not obligate phenotypes in blood-derived chromosomes, even when pericentromeric and centromeric repeats are hypomethylated. However, ICF-typical cytogenetic abnormalities may appear in patient-derived LCLs, even when they are missing in the blood derived chromosomes. Interestingly, we detected in the patient’s blood a significant elevation in premature sister chromatid separation (Supplementary Fig. 2A), a phenotype that was not reported previously in any ICF syndrome patient. It was previously suggested that DNA methylation may affect chromosome compaction and sister chromatid cohesion [44, 45], and cohesion binding is reduced following DNA hypomethylation at the dispersed Alu repeats [46]. However, since chromatin localization of SMC3 is not affected in DNMT triple knock-out cells [47] the involvement of DNA methylation in sister chromatid cohesion remains to be examined. Intriguingly, in yeast, downregulation of Irc5, the orthologue of *HELLS*, the gene responsible for ICF4, induces premature chromatid separation [48]. In human cells, the cohesion components SMC1A and SMC3 were identified as proteins that require CDCA7 to accumulate on newly synthesized DNA [49]. CDCA7 is known to form a chromatin remodeling complex with HELLS [29, 50], and comprehensive mass spectrometric analyses have identified that HELLS potentially interacts with several components of the cohesin complex, including SMC1A, SMC3, and SMC5 [51–53]. Thus, a reduced amount of CDCA7 caused by the BTB pathogenic variant could lead to premature sister chromatid separation, possibly by impairing the function of the CDCA7/HELLS complex and directly hampering the cohesion. Additional abnormalities in centromeric functionality, recently revealed in ICF2 patients [27], indicate that ZBTB24 protects centromeric integrity through several mechanisms. All these support the notion that DNA methylation at centromeres is crucial for centromeric integrity, and suggest that premature sister chromatid separation in human chromosomes may also arise as a consequence of centromeric/pericentromeric DNA hypomethylation.

Immunodeficiency, another hallmark phenotype apparent in ICF syndrome, is very variable among ICF2 patients, including among siblings carrying identical *ZBTB24* variants [54]. A survey of 39 ICF2 patients demonstrated that abnormally low levels of IgM were apparent in 90% of the patients, and normal levels of IgG and IgA were found in 20% and 25% of patients, respectively [20]. Other studies also reported normal IgA and IgG levels in ICF2 patients [21, 22]. In some cases, the immunodeficiency developed gradually over several years [54, 55]. In RZ, despite the severe infections she experienced initiating at her first year of life, her IgA and IgG levels were normal and she responded to her routine vaccinations during the first five years of life. However, throughout this period, her IgM levels were intermittently below normal or at the lower limit of the normal range (Table 1). Based on the data we collected, we speculate that RZ was prone to invasive pneumococcal disease at infancy chiefly because of SCD [56]. The patient was found to suffer from complete functional asplenia, which seems to be the main factor responsible for her severe pneumococcal infections [57]. Her clinical presentation is in line with the literature regarding SCD [58], including complete functional asplenia, which is common in patients with Hb SS SCD [59]. The lack of sonographic findings related to splenic infarctions may be attributed to the packed red blood cell transfusions and the folic acid and hydroxyurea treatment RZ received [60]. However, we cannot rule out that her immunodeficiency results from a combined effect of SCD and ICF syndrome. To note, this is the first reported patient with ICF2 and functional asplenia, as well as the first patient to have SCD in concurrence with ICF2.

RZ represents the first reported ICF2 patient with biallelic missense variants in the BTB domain of ZBTB24. The BTB domain is a protein–protein interaction domain which drives homodimerization, and less frequently heterodimerization with other ZBTB family members, and interactions with additional proteins that do not contain BTB domains [25]. Specifically for ZBTB24 (also known as PATZ2), in addition to homodimerizing, the BTB domain can heterodimerize with the BTB domain of ZBTB19 (PATZ1) [40]. Therefore, amino acid changes in the BTB domain could potentially disrupt such interactions. Previously the generation of ZBTB homodimers or heterodimers was studied on isolated BTB domains, and not in the context of full-length ZBTB proteins [40]. In this study we validated homodimerization of the full-length WT ZBTB24 (Fig. 4A). While interrogating whether the p.V43L variant disrupts dimer formation, we uncovered that this variant actually decreases full-length ZBTB24. This is true for both exogenously expressed FLAG-ZBTB24-V43L (Fig. 4A and B), and for the endogenous ZBTB24-V43L protein expressed in patient RZ LCLs (Fig. 4D). Importantly, an additional rare missense BTB domain variant, p.S59G, reported in a compound heterozygote ICF2 patient [33], also leads to a decrease in full-length FLAG-tagged ZBTB24 (Supplementary Fig. 4). In contrast, a missense variant, p.R49Q (rs147441359), also positioned in the BTB domain, does not destabilize ZBTB24 when incorporated into an exogenously expressed TY1-flagged ZBTB24 [34].

A question arises regarding the pathway by which the BTB pathogenic missense variants result in ZBTB24 degradation. Proteasome mediated degradation is a possible mechanism in this case, as defects in dimerization of BTB containing-proteins could lead to rapid proteasome-dependent protein degradation due to exposure of a degron/s positioned in the BTB domain [38]. This mechanism, termed dimerization quality control (DQC) guarantees that only functional BTB-containing proteins will be present in cells. Specifically for ZBTB proteins, three degron sequences were identified in the BTB domain [38]. Interestingly, however, AlphaFold 3, which also predicts homodimerization of full length WT ZBTB24 via its BTB domain (pTM = 0.71, iPTM = 0.71), does not place the two relevant amino acids, V43 and S59, on the surface of the interaction sites of the dimer (Supplementary Fig. 8). An additional study similarly suggests that positions V43 and S59 are not crucial for the formation of the homodimer interface [40]. Thus, it is possible that the V43 and S59 substitutions cause protein degradation by destabilization of the protein structure rather than by directly preventing dimerization. The novel ~ 50 kDa band interacting with the ZBTB24 antibody directed to the C-terminus of ZBTB24, detected in the patient and her mother’s LCLs (Fig. 4D), suggests a process of degradation initiating from the N-terminus of the protein but failing to processively degrade the whole protein, a phenomenon occasionally seen in proteasome substrates [61–64]. If such is the case, the N-terminus FLAG tag could have contributed to the relative stability of the exogenously expressed ZBTB24 compared to the endogenously expressed protein by inhibiting the initiation of degradation (Fig. 4A, B and Supplementary Fig. 4). To interrogate the involvement of the proteosome, we tested whether known proteasome inhibitors stabilize the mutant ZBTB24 proteins, but detected only very limited stabilization of both endogenously and exogenously expressed ZBTB24 (Supplementary Fig. 6). Thus, we cannot confirm unequivocally that the degradation of BTB-mutated ZBTB24 occurs through the proteasome, although we also cannot completely rule out this mechanism. We also could not find evidence for degradation of the mutated ZBTB24 through the autophagy pathway. At any rate, the strong evolutionary conservation of these two amino acids among many organisms (Supplementary Fig. 9), and between many different ZBTB proteins [39], which is not apparent for R49, suggests that they are crucial for the functionality of the BTB domain.

Pathogenic variants in *ZBTB24* are associated with reduced transcription of *CDCA7*, one of its targets. and the causative gene for ICF3 syndrome [9, 12]. As expected, *CDCA7* mRNA levels in patient RZ LCLs were significantly reduced compared to those detected in WT or SZ (heterozygote) LCLs (Fig. 4E). Additional roles of ZBTB24, such as involvement in class switch recombination [28], indicate that the abnormal phenotypes in ICF2 syndrome are mediated not only through defective *CDCA7* activation. The striking changes in DNA methylation in the genomes of ICF2 patients affect the expression of many genes [27], and further studies will have to elucidate the pathways by which the various phenotypes of ICF2 patients, as well as the additional ICF types, are brought upon.

In this study we demonstrate for the first time that biallelic missense variants in the BTB domain of ZBTB24 can also function as causative variants in ICF2 syndrome. The mechanism of pathogenicity of the studied BTB variants is LOF, similar to the other pathogenic variants previously reported in *ZBTB24*. Acknowledging new domains that can harbor pathogenic variants, especially of missense type, will hopefully enhance the identification of additional patients inflicted with the rare ICF2 syndrome, and further elucidate the clinical phenotype spectrum and molecular basis of the disease.

## Materials and methods

### Patient and family members

Consent was obtained from participants in the study, including photo consent. Peripheral blood samples were taken under a research protocol approved by the Israeli Ministry of Health Ethics Committee (protocol number 920110505).

### Tissue culture and generation of lymphoblastoid cell lines

LCLs were grown in RPMI supplemented with 20% fetal bovine serum, glutamine and antibiotics. pD LCLs were derived from a previously described ICF2 patient [35]. A control LCL, GM18486, was purchased from Coriell Institute for Medical Research. RZ and SZ LCLs were generated from peripheral blood leukocytes by EBV infection [65]. Human embryonic kidney 293 T (HEK293T) cells were cultured in Dulbecco's Modified Eagle's Medium supplemented with 10% FBS and antibiotics.

### Cell transfections

HEK293T cells were transfected with human wild-type (WT) or mutant *ZBTB24*-expressing plasmids (see below) using PolyJet transfection reagent (SL100688, SignaGen Laboratories) or FuGene HD transfection reagent (E2311, Promega), according to the manufacturer’s instructions. Forty-eight hours following transfection cells were harvested, and pellets were prepared for RNA and protein extractions.

### Treatment of cells in culture with inhibitors

To block the proteasome, cells were treated with 20 μM MG132 (A2585, APExBIO), or with 0.5 μM Bortezomib (179324–69-7, Merck Calbiochem) for six hours prior to harvest. To block autophagy, cells were treated with either 5 nM or 10 nM Bafilomycin A1 (SML1661, Sigma-Aldrich) for 18 h prior to harvest. Protein extracts were prepared for western blot analysis as described below.

### Chromosome preparation

Metaphase spread preparations from peripheral blood leukocytes and LCLs and G-banding were performed using standard procedures. Capture and analysis of metaphase images were done with an Applied Spectral Imaging system (Migdal HaEmek, Israel).

### Whole exome and Sanger sequencing

Exome sequencing was conducted at CeGaT GmbH (Tübingen, Germany) on a Novaseq6000 platform (Illumina, San Diego, CA) using the Twist Exome Capture Enrichment kit (Twist Bioscience HQ, South San Francisco, CA). Mapping of the obtained reads to the reference genome (build GRCh37/hg19), variant calling, annotation, and data analysis were done using the Genoox data analysis platform Ltd (Palo Alto, CA).

Sanger confirmation of variants detected by exome analysis and segregation studies were performed according to standard methods. The following primers were utilized to amplify the variant-containing region, localized to exon 2 (*ZBTB24* MANE transcript, RefSeq NM_014797.3)-ZBTB24-Ex2-For: 5′-CCTTAGTGGCTTCTGAAG-3′ and ZBTB24-Ex2-Rev: 5′-GCATGGAGATAACCTGTG-3′.

### Site directed mutagenesis (SDM)

*ZBTB24* c.127G > C and c.175A > G variants were separately introduced by site directed mutagenesis (SDM) into a p3xFLAG-CMV-10 plasmid containing *ZBTB24*_WT_ cDNA Open Reading Frame [66]. (generating pFLAG-ZBTB24_V43L_ and pFLAG-ZBTB24_S59G_, respectively). SDM was performed utilizing PfuUltra II Fusion hot start DNA polymerase (#600672–51, Agilent), and the following primers (variant inserted in the process is underlined): for c.127G > C (Forward: 5′-*TCTGTGACATTACTTTAATCCTGGAGAATGTACATTTCCGG*-3′ and Reverse: 5′-*CCGGAAATGTACATTCTCCAGGATTAAAGTAATGTCACAGA*-3′), and for c.175A > G (Forward: 5′-*AGCCTTACTTGCTGCCAGTGGTGAATACTTCTCAATGAT*-3′ and Reverse: 5′-*ATCATTGAGAAGTATTCACCACTGGCAGCAAGTAAGGCT*-3′). Positive clones were sequenced along the entire cDNA and the FLAG region to verify that no additional changes were incorporated during the SDM process.

### DNA methylation analysis by bisulfite conversion, TA cloning and Sanger sequencing

Genomic DNA was extracted from peripheral blood leukocytes using the FlexiGene DNA kit (51 206, QIAGEN). DNA from LCLs was extracted using Cells and Tissue Isolation kit (#53100, Norgen Biotek Corp.). 50–200 ng of DNA were bisulfite-converted using the Methylamp DNA modification kit (P-1001-1. EPIGENTEK, NY). Bisulfite converted DNA was amplified using Ex Taq DNA polymerase (RR001, TaKaRa) as follows: 95°C-5 min; 4 cycles of: 95°C-1 min, low annealing temperature-54°C-3 min, 72°C-3 min; 40 cycles of: 95°C-30 s, high annealing temperature-57°C 45 s, 72°C- 45 s; 72°C-10 min.

Bisulfite-converted primer sequences utilized included: sat 2 For: 5′-AATGAAAGGAGTTATTATTTAATGG-3′ and sat 2 Rev: 5′-CATTCCATTCCATTAAATAATTCC3′ (unconverted sequences: 5′-AATGAAAGGAGTCATTATCTAATGG-3′ and 5′-CATTCCATTCCATTAGATGATTCC-3′, respectively). PCR amplicons are located to the hsat2_1_3(A1,B), hsat2_10_2(A1,A2) and hsat2_16_15(B) satellite repeats, as annotated in the T2T CHM13v2.0/hs1 release. PCR products were TA-cloned into pCR2.1 plasmid (K2020, Invitrogen). Inserts were Sanger sequenced using M13 or T7 universal primers.

### Whole genome DNA methylation profiling

Bisulfite-converted DNA was subjected to genome-wide methylation profiling using Infinium Methylation EPIC 850 K Bead Chip (Illumina Inc., USA). Fluorescence signal intensities were captured using Illumina HiScan SQ (Illumina Inc., USA). We analyzed the data in R version 4.1.0 through the ChAMP Bioconductor package (v 2.21.1) [67]. Using the ‘champ.load’ function, we imported the raw idat files in R to calculate the β-values with quality control and filtering options set as default and array type as ‘EPIC’. To normalize the methylation signal coming from Type I and Type II probes, we applied the BMIQ normalization using the ‘champ.norm’ function with default parameters and array type as ‘EPIC’. Further, to highlight the methylation defects of the patient blood, we downloaded peripheral blood controls and ICF2 patient data [12] and took the common probes between the array using the combineArray function in minfi Bioconductor package v1.40.0 [68]. Using a linear model built with ‘limma’ package [69], we calculated the differentially methylated CpG sites with a mean β-values difference > =|0.30| and adjusted *P*-value < 0.05. Using the DMPs, we extracted the associated genes and performed the pathway analysis with g:Profiler (Benjamini-Hochberg FDR, *P*-adj < 0.05). To analyze the repetitive regions, we downloaded the coordinates from UCSC (http://hgdownload.cse.ucsc.edu/goldenpath/hg19/database/rmsk.txt.gz) and annotated the CpGs to the different satellite repeat families. Satellite repeats covered by at least 10 probes were considered for analysis (Supplementary Table 1). Statistical significance of DNA methylation changes was calculated using a two-tails Wilcoxon test with Bonferroni correction. Significantly different samples are indicated as follows: (*) *P*-value < 0.05, (**) *P*-value < 0.01, (***) *P*-value < 0.001, (****) *P*-value < 0.0001.

### Data access

Illumina EPIC array raw and processed data are deposited in the GEO repository under accession GSE249219.

### Reverse transcription quantitative PCR (RT-qPCR)

RNA was isolated using the RNeasy Mini Kit (74 104, QIAGEN). cDNA was prepared from 2 μg of RNA using the 5X All-in-One MasterMix (G485, Applied Biological Material, Inc.). cDNA was diluted 1:10 and RT-qPCR was performed in triplicates using Fast SYBR Green Master Mix (4 385 612, ThermoFisher SCIENTIFIC), on a StepOnePlus™ Real-Time PCR System. Expression levels were normalized to the *β-actin* (*ACTB*) gene using the 2^-ΔΔ*C*T^ method [70]. Primers used for amplification were based on the MANE transcripts as following: *ZBTB24* (Forward: 5′-*TCATTACCGGAATGCAAAGA*-3′ (exon 5) and Reverse: 5′-*TGGATTCTGATGTGGGTCTG*-3′ (exon 6)), *CDCA7* (Forward: 5′-*AACGTCTGCAGCAATTCTCG*-3′ (exon 7) and Reverse: 5′-*AGCATCCCTGACCTCTTCAC*-3′ (exon 8)), *RNF187* (Forward: 5′-*GGAGAACAAGGGGTCTGTGG*-3′ (exon 2) and Reverse: 5′- GTGCCTTCTTCCTACGGTCC-3′ (exon 3)), *ACTB* (Forward: 5′-*TGTACGCCAACACAGTGCTG* − 3′(exon 5) and Reverse: 5′-*GCTGGAAGGTGGACAGCGA* − 3′ (exon 6)), and exogenously expressed FLAG-ZBTB24 (Forward on FLAG-tag: 5′-*GACTACAAAGACCATGACGG*-3′ and Reverse on *ZBTB24*: 5′-*TCAAAACTGGCCAGCACAG*-3′ (exon 2)). The ZBTB24 genotype in each cDNA sample was confirmed by Sanger sequencing.

### Protein extraction and western blot analysis

Cells were washed three times with phosphate buffered saline (PBS) and resuspended in 120 μL of extraction buffer (20 mM Tris–HCl (pH 7.4), 150 mM NaCl, 0.5% NP40, containing protease inhibitor cocktail tablets (11 697 498 001, MERCK), following sonication at 6°C for 3 min, 200 cycles/burst (M220 Focused-ultrasonicator, Covaris). Protein lysates were quantified by Bradford protein assay (5 000 006, Bio-Rad). An equal volume of 5X non reducing lane marker sample buffer (39 001, Thermo Scientific Pierce) containing 40 mM DTT was added, and samples were boiled for 5 min. 60 μg of total protein lysates were separated by SDS polyacrylamide gel electrophoresis on a 4–20% gradient gel (4561094–4-20, Bio-Rad) transferred to Whatman Protran BA83 nitrocellulose membranes (WHA10401316, Merck), and blocked with 5% skim milk in TBST at room temperature. ZBTB24 was detected with guinea pig polyclonal anti-ZBTB24 antibody (PM086, MBL, 1:1000) and HRP-conjugated anti-guinea pig IgG (H + L) (106–035-003, Jackson ImmunoResearch Inc., 1:5000). FLAG-tagged proteins were detected with mouse anti-FLAG M2 antibody (F1804, Sigma-Aldrich, 1:1000). Membranes were incubated in WesternBright ECL (K-12045-D20, Advansta Inc.) 2 min, and exposed in a Fusion FX apparatus (Vilber). For protein loading normalization, membranes were also reacted with an anti-β-actin rabbit polyclonal antibody (A2066, Sigma-Aldrich, 1:5000). LC3 was detected with an anti LC3 mAb-HRP-DirecT antibody (M186–7, MBL, 1:1000).

### Dimer analysis by immunoprecipitation (IP)

HEK293T cells were co-transfected with the following plasmid combinations: pMyc-ZBTB24_WT_ with pFLAG-ZBTB24_WT_; pMyc-ZBTB24_WT_ with pFLAG-ZBTB24_V34L_; and pMyc-ZBTB24_WT_ with pMax_GFP. pMax_GFP was a gift from Zachary Nagel & Leona Samson (Addgene plasmid # 177825; http://n2t.net/addgene:177825; RRID:Addgene_177 825) [71]. Forty-eight hours after transfection, cells were harvested, lysed in 0.5% NP-40 lysis buffer (150 mM NaCl, 0.5% NP-40 and 50 mM Tris–HCl, pH 8.0), homogenized by sonication, and incubated on ice for 30 min. The supernatant was collected by centrifugation. Precleaning was performed with the addition of protein A/G PLUS-Agarose (sc-2003, Santa Cruz Biotechnology) and normal mouse IgG (sc-2025, Santa Cruz Biotechnology) to the supernatant. After rotation for 1 hour at 4°C, the precleaned samples were collected, and immunoprecipitation was performed using anti-FLAG M2 Affinity Agarose Gel (A2220, Sigma-Aldrich) for 1 hour at 4°C. After washing the agarose with the lysis buffer 5 times, immunoprecipitated proteins were eluted using 3 × FLAG peptides (F4799, Sigma-Aldrich) and subjected to western blot analysis. The immunoprecipitated proteins were detected by anti-FLAG rabbit polyclonal antibody (PM020, MBL, 1:1000) or anti-Myc mouse monoclonal antibody (M192–3, MBL, 1:1000). After several washes, blots were incubated with HRP-conjugated anti-rabbit or mouse IgG antibody (ab6789 or ab6721, abcam, 1:30000), and detected by using Chemi-Lumi One Ultra reagent (11644–40, Nacalai tesque, Japan) and iBright FL1500 Imaging System (Life Technologies).

### Prediction of the homodimer structure of the ZBTB24 protein

We employed AlphaFold3 (https://alphafoldserver.com/) to predict the three-dimensional structure of the ZBTB24 protein in its homodimer form via the BTB domain [72]. PyMOL software (version 3.1.0, https://pymol.org/) was used to generate an illustrative image.

## Supplementary Material

Supplementary_Figures_Givol_et_al_051125_ddaf182-2

Revised_Supplementary_Table_1_Givol_et_al_ddaf182
