# Supplementary material for: Missense substitutions in the BTB domain of ZBTB24 can lead to protein instability and cause ICF2 syndrome
Source: Hum Mol Genet. 2025 Dec 8;35(2):ddaf182. doi: 10.1093/hmg/ddaf182 (PMC13158236; doi:10.1093/hmg/ddaf182)
Supplement: Supplementary_Figures_Givol_et_al_051125_ddaf182-2 [file supplementary_figures_givol_et_al_051125_ddaf182-2.pdf]

Supplementary Figures

A

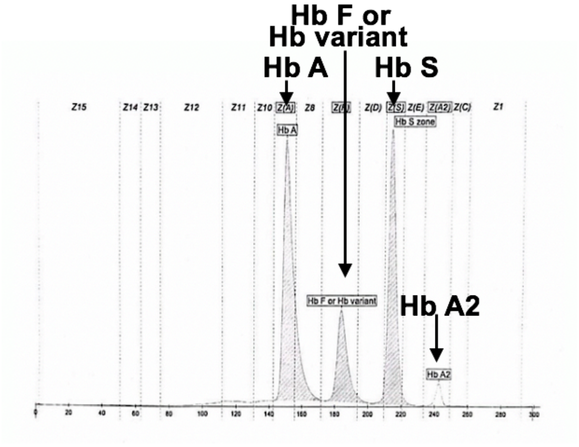

Hemoglobin electrophoresis

Hb type	%	Ref. %
Hb A	46.7	96
Hb F or Hb variant	16.0	0.6-2
Hb S zone	35.1	0
Hb A2	2.2	1.5-3.7

B

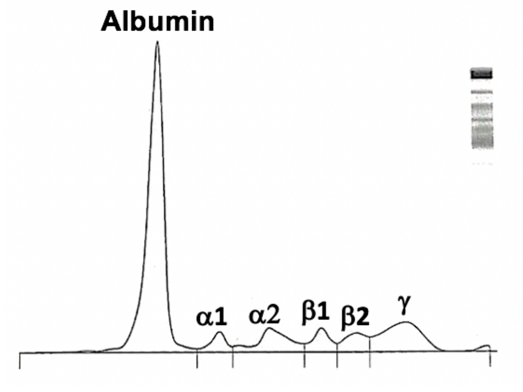

Serum protein electrophoresis

Fraction	%	Ref. %
Albumin	62.4	55.8-66.1
Alpha 1 (α1)	3.8	2.9-4.9
Alpha 2 (α2)	8.9	7.1-11.8
Beta 1 (β1)	4.9	4.7-7.2
Beta 2 (β2)	5.2	3.2-6.5
Gamma (γ)	14.8	11.1-18.8

Supplementary Figure 1

Hemoglobin electrophoresis and serum protein electrophoresis.

A. Hemoglobin (Hb) electrophoresis of patient RZ's blood. The normal Hb variants present in adult hemoglobin are indicated by the Hb A and Hb A2 peaks. The Hb F peak indicates fetal Hb and should normally constitute up to 2% of the total Hb variants. The value of Hb F in patient RZ is significantly higher due to treatment with Hydroxyurea. Hb S notes the sickle cell Hb and is present only in individuals who are heterozygous or homozygous for the sickle cell disease mutation in *HBB*. The electrophoresis shown in A was performed shortly after the patient received a dose of packed red blood cells due to symptomatic anemia, explaining why the Hb S constitutes only 35% of the various Hb variants. **B.** Serum protein electrophoresis. Normal distribution of the serum proteins was noted with no evidence for monoclonality in the gamma region. The α2 peak is close to the lower limit of the normal range most likely due to low levels of Haptoglobin, which are common in patients with ongoing hemolysis. The β1 peak is also close to the lower limit of the normal range due to low Transferrin levels secondary to the high Ferritin levels resulting from repetitive doses of packed red blood cells. The serum electrophoresis was performed during a routine blood sampling and not during a severe hemolytic event.

A

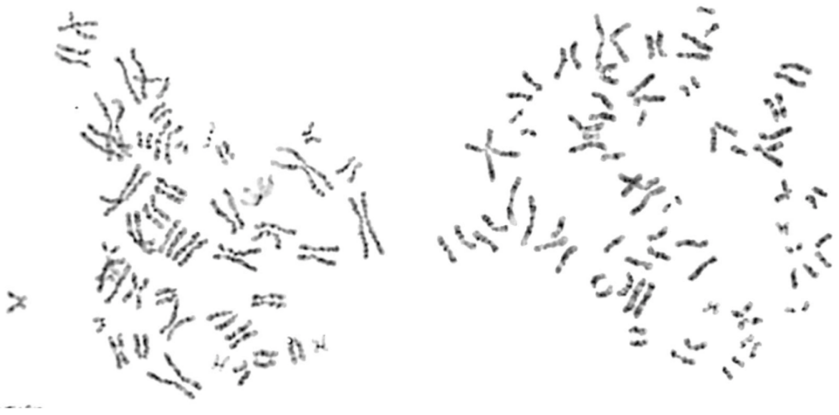

Sample	PSCS	Total metaphases	*P*-value
**RZ**	**8**	**117**	-
**WT1**	**0**	180	$4.99x10^{-4}$
**WT2**	**0**	132	$2.08x10^{-3}$
**WT3**	**0**	158	$9.31x10^{-4}$
**WT - total**	**0**	470	$2.04x10^{-6}$

B

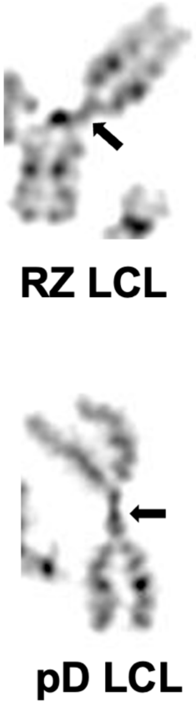

Supplementary Figure 2

Cytogenetic phenotypes in RZ's blood and LCLs

A. Premature sister-chromatid separation is elevated in patient RZ's blood. Cytogenetic analysis of patient RZ's white blood cells revealed metaphase spreads with premature sister-chromatid separation (PSCS) at a rate of 6.8% (8 out of 117). The top panel displays two typical metaphase spreads from RZ's blood showing PSCS in the vast majority of chromosomes. Analysis of over 100 metaphase spreads from RZ's blood and each of three WT controls demonstrates that the levels of PSCS in RZ are significantly higher. Analysis was done by Fisher's exact test.

B. LCLs derived from RZ demonstrate an ICF-typical cytogenetic phenotype. Cytogenetic analysis of RZ LCLs revealed decondensation of the pericentromeric region of one copy of chromosome 1 in 7.7% of the examined metaphase spreads (eight out of 103). Analysis of pD LCLs demonstrated a similar phenotype in 11.8% (13 out of 110) metaphase spreads. A representative example of chromosome 1 from each LCLs is displayed in **B**. The arrow points to the pericentromeric region.

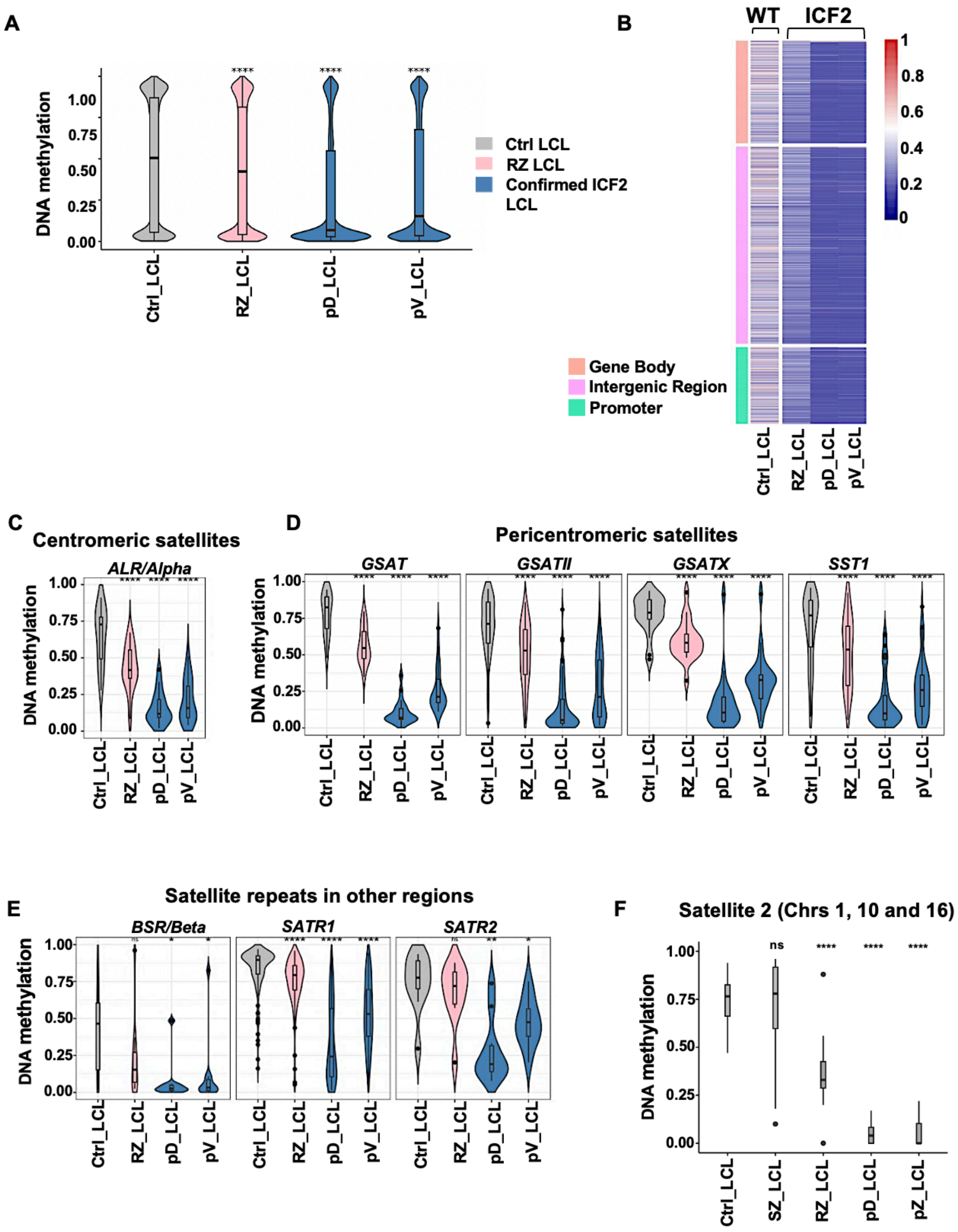

Supplementary Figure 3

Whole genome DNA methylation profiling of RZ LCLs DNA demonstrates significant hypomethylation, but to a lesser degree compared to other ICF2 patient LCLs

A. A violin plot showing the distribution of whole-genome DNA methylation levels in control (ctrl), RZ, and ICF2 pD and pV LCLs. **B.** A heatmap depicting the DNA methylation levels of 2183 DMPs in control, RZ and ICF2 pD and pV LCLs. The genomic features of DMPs, i.e gene body, intergenic region or promoter associated, are highlighted as row annotations. **C. - E.** Violin plots showing the distribution

of methylation levels of Satellite repeats localized to (C.) centromeric (ALR/alpha), (D.) pericentromeric (GSAT, GSATII, GSATX, SST1) and (E.) other genomic regions (BSR/Beta, SATR1, SATR2) in control, RZ and ICF2 pD and pV LCLs. **F.** Boxplot displaying DNA methylation levels of satellite 2 (sat 2) repeats specific to chromosomes 1, 10 and 16, measured by bisulfite sequencing in LCLs derived from RZ patient, RZ's healthy mother SZ, ICF2 pV and pD and control LCL. *P*-adjusted values represent the Bonferroni-corrected *p*-values obtained from a two-sample Wilcoxon test with two-sided alternatives. (∗) p-value < 0.05, (∗∗) p-value < 0.01, (∗∗∗) p-value < 0.001, (∗∗∗∗) p-value < 0.0001.

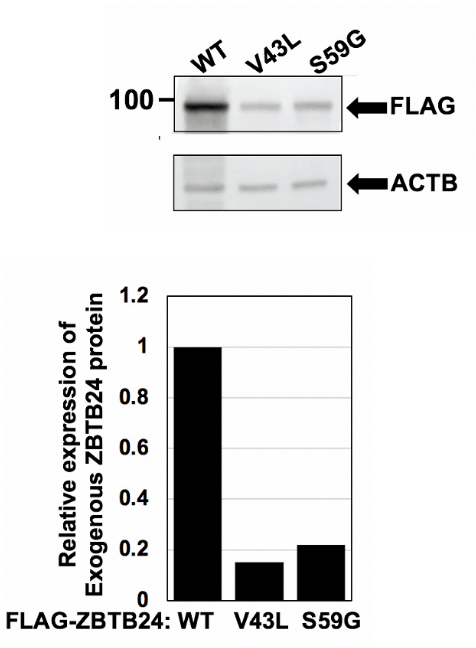

Supplementary Figure 4

BTB missense variants V43L and S59G destabilize ZBTB24 protein.

Top - HEK293T cells were transfected with each of the following plasmids: pFLAG-ZBTB24$_{WT}$, pFLAG-ZBTB24$_{V43L}$, or pFLAG-ZBTB24$_{S59G}$. Forty-eight hours post-transfection, proteins were extracted from cells and western blot analysis was performed. Accurate protein loading was verified by using β-actin (ACTB) protein levels as a control. The 100 kDa size marker appears on the left. Bottom - Signal intensities of the FLAG-ZBTB24$_{V43L}$ and FLAG-ZBTB24$_{S59G}$ bands obtained by Western blot analysis (top) were normalized to that of FLAG-ZBTB24$_{WT}$.

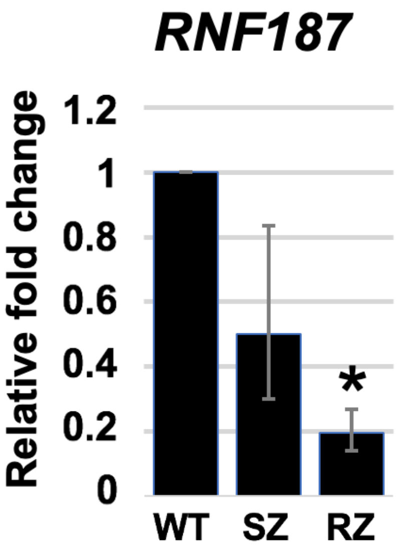

Supplementary Figure 5

The mRNA expression level of RNF187 is significantly downregulated in RZ LCLs

Expression levels of *RNF187,* a transcriptional target of ZBTB24, a were assessed in WT (control GM18486), SZ and RZ LCLs by RT-qPCR, normalized to β-actin and presented as fold change relative to expression levels in WT LCLs. RT-qPCR was performed in biological triplicates. Error bars represent standard errors. p-values were determined by Student's t-test (* - p<0.05, ** - p<0.01, *** - p<0.001).

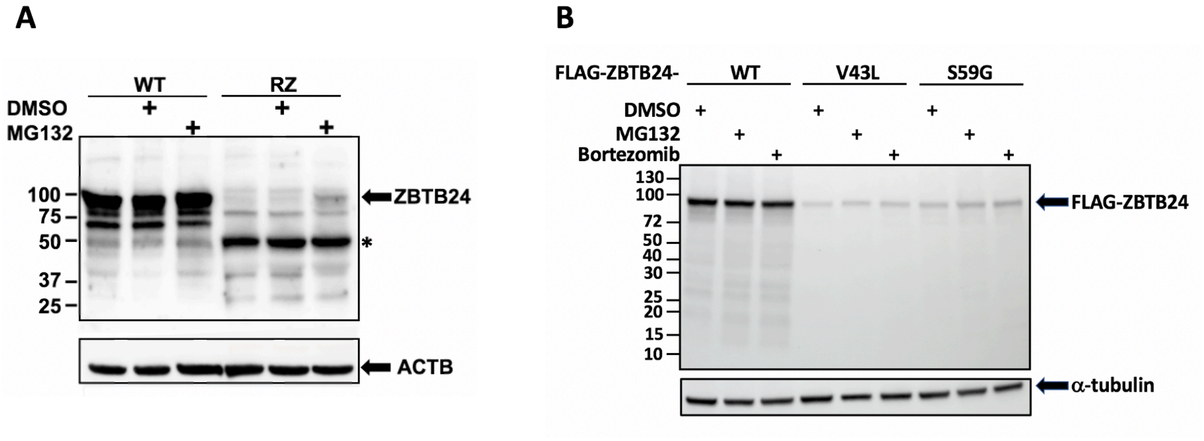

Supplementary Figure 6

Proteasome inhibitors do not substantially stabilize ZBTB24 proteins containing pathogenic BTB-missense variants. A. Proteins extracted from control (WT) and patient RZ LCLs untreated or treated with DMSO or MG132, were subjected to western analysis. While $ZBTB24_{WT}$ was stabile under all conditions, endogenous $ZBTB24_{V43L}$ was unstable, as demonstrated in Figure 4D, and was stabilized to a very low degree following treatment with MG132. Accurate protein loading was verified using β-actin (ACTB) protein levels as a control. Asterisk marks a possible degradation product. Size markers in kDa appear on the left. **B.** HEK293T cells were transfected with each of the following plasmids: pFLAG-$ZBTB24_{WT}$, pFLAG-$ZBTB24_{V43L}$ or pFLAG-$ZBTB24_{S59G}$. Six hours before harvest (and 48 hours post transfection), cells were treated with DMSO, MG132 or Bortezomib. Proteins were extracted from cells and western analyses was performed with and anti-FLAG antibody to detect only the exogenous ZBTB24 protein. Accurate protein loading was verified by α-tubulin protein levels. Size markers in kDa appear on the left. FLAG-$ZBTB24_{WT}$ was stable under all conditions. Proteasome inhibitors did not stabilize the exogenously expressed ZBTB24 proteins containing the BTB missense variants.

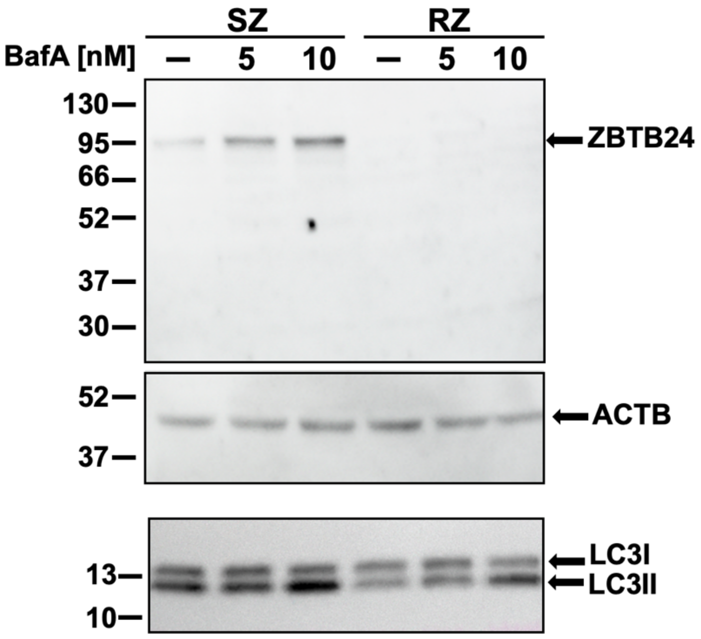

Supplementary Figure 7

Blocking of autophagy does not stabilize ZBTB24 in patient RZ LCLs.

SZ and RZ LCLs were treated for 18 hours with two concentrations of Bafilomycin A1 (BafA), to block autophagy. While ZBTB24 levels were elevated moderately in LCLs of SZ, the mother of RZ, there is no evidence for stabilization of the protein in patient RZ's LCLs. The stabilized protein in SZ LCLs may be derived from the WT *ZBTB24* allele of SZ. Accurate protein loading was verified using β-actin (ACTB) protein levels as a control. Protein extracts were reacted also with an anti-LC3 antibody. The accumulation of LC3II following treatment with BafA indicates that autophagy was blocked. Interestingly, the degradation product of approximately 50kD shown in the patient cells in other experiments is not visible here.

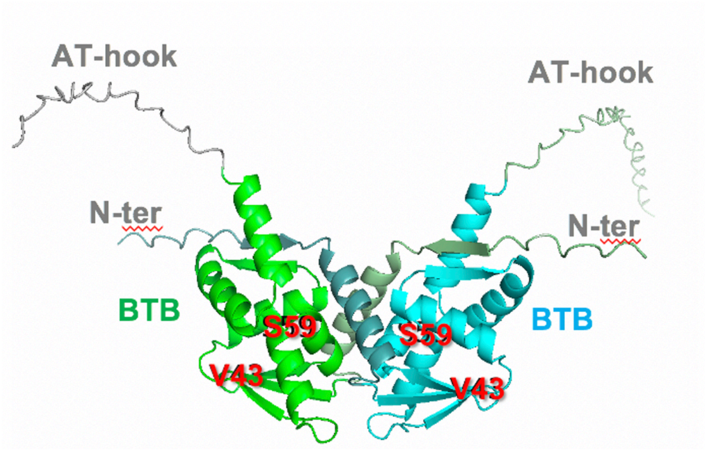

Supplementary Figure 8

Prediction of the homodimer structure of the ZBTB24$_{WT}$ protein. A three-dimensional structure of the ZBTB24$_{WT}$ protein (residues 1-171, including the BTB domains and AT-hook motif) forming a homodimer via the BTB domain, was predicted using AlphaFold3. PyMOL software was used to generate the illustrative image, highlighting the locations of V43 and S59.

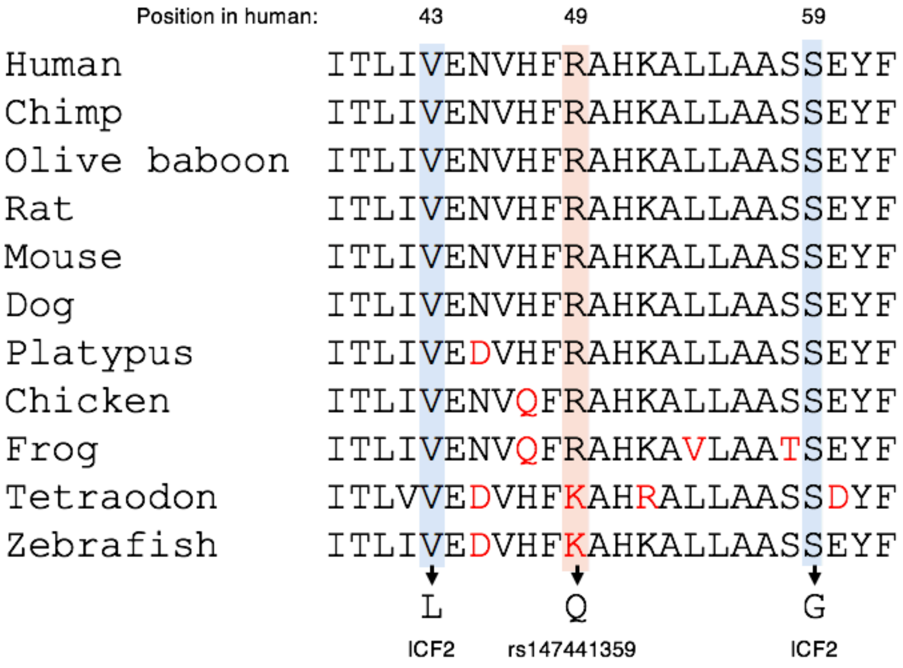

Supplementary Figure 9

Strong evolutionary conservation of V43 and S59, but not of R49, in ZBTB24 protein

The two pathogenic missense variants identified in the BTB domain of ZBTB24, V43L and S59G, (position depicted by the blue strips) are highly conserved amino acids in ZBTB24. In contrast, the R49Q variant (frequency: 1691/251485 = 0.006724, GnomAD_exome), (position depicted by the pink strip), was demonstrated to be benign (Wu et al. 2019).
